# Comedonecrosis Gleason pattern 5 is associated with worse clinical outcome in operated prostate cancer patients

**DOI:** 10.1038/s41379-021-00860-4

**Published:** 2021-06-26

**Authors:** Tim Hansum, Eva Hollemans, Esther I. Verhoef, Chris H. Bangma, John Rietbergen, Susanne Osanto, Rob C. M. Pelger, Tom van Wezel, Henk van der Poel, Elise Bekers, Jozien Helleman, Sebastiaan Remmers, Geert J. L. H. van Leenders

**Affiliations:** 1 Department of Pathology, Erasmus MC, University Medical Center, Rotterdam, The Netherlands; 2 Department of Urology, Erasmus MC, University Medical Center, Rotterdam, The Netherlands; 3 Department of Urology, Franciscus Gasthuis & Vlietland Hospital, Rotterdam, The Netherlands; 4 Department of Medical Oncology, Leiden University Medical Center, Leiden, The Netherlands; 5 Department of Urology, Leiden University Medical Center, Leiden, The Netherlands; 6 Department of Pathology, Leiden University Medical Center, Leiden, The Netherlands; 7 Department of Urology, Netherlands Cancer Institute, Amsterdam, The Netherlands; 8 Department of Pathology, Netherlands Cancer Institute, Amsterdam, The Netherlands

**Keywords:** Prostate cancer, Prostate cancer

## Abstract

Individual growth patterns and cribriform architecture are increasingly considered in risk stratification and clinical decision-making in men with prostate cancer. Our objective was to establish the prognostic value of individual Gleason 5 patterns in a radical prostatectomy (RP) cohort. We reviewed 1064 RPs and recorded Grade Group (GG), pT-stage, surgical margin status, Gleason 4 and 5 growth patterns as well as intraductal carcinoma. The clinical endpoints were biochemical recurrence and post-operative distant metastasis. Gleason pattern 5 was present in 339 (31.9%) RPs, of which 47 (4.4%) presented as primary, 166 (15.6%) as secondary, and 126 (11.8%) as tertiary pattern. Single cells/cords were present in 321 (94.7%) tumors with Gleason pattern 5, solid fields in 90 (26.5%), and comedonecrosis in invasive carcinoma in 32 (9.4%) tumors. Solid fields demonstrated either a small nested morphology (*n* = 50, 14.7%) or medium to large solid fields (*n* = 61, 18.0%). Cribriform architecture was present in 568 (53.4%) RPs. Medium to large solid fields and comedonecrosis coincided with cribriform architecture in all specimens, and were not observed in cribriform-negative cases. In multivariable analysis adjusted for Prostate-Specific Antigen, pT-stage, GG, surgical margin status and lymph node metastases, cribriform architecture (Hazard Ratio (HR) 9.9; 95% Confidence Interval (CI) 3.9–25.5, *P* < 0.001) and comedonecrosis (HR 2.1, 95% CI 1.2–3.7, *P* = 0.01) were independent predictors for metastasis-free survival, while single cells/cords (HR 1.2; 95% CI 0.7–1.8, *P* = 0.55) and medium to large solid fields (HR 1.6, 95% CI 0.9–2.7, *P* = 0.09) were not. In conclusion, comedonecrosis in invasive carcinoma is an independent prognostic Gleason 5 pattern for metastasis-free survival after RP. These data support the current recommendations to routinely include cribriform pattern in pathology reports and indicate that comedonecrosis should also be commented on.

## Introduction

The Gleason grading system has a strong predictive value for clinical outcome in prostate cancer patients and is entirely based on architectural growth pattern assessment [1]. According to the 2014 International Society of Urological Pathology (ISUP) consensus meeting, growth patterns are categorized in three groups; Gleason pattern 3, 4, and 5 [2, 3]. In radical prostatectomy (RP) specimens, the two most common Gleason patterns are added, resulting in a score of 6–10. Whereas Gleason score 2–5 can be assigned on operation specimens, their distinction from Gleason score 6 has no clinical relevance. Men with Gleason score ≤6 (Grade Group 1) on RP have excellent outcome with no or very low risk of developing metastatic disease [4–7]. Risk of biochemical recurrence, metastasis and disease-specific mortality increments with higher Gleason scores [8–13].

Gleason pattern 4 includes at least four different growth patterns which are currently classified as fused, poorly formed, glomeruloid and cribriform glandular structures. Last decade it has become clear that cribriform growth pattern is associated with adverse clinical outcome, being an independent predictive factor for biochemical recurrence, metastasis and disease-specific death [14–18]. Therefore, the latest 2019 ISUP consensus meeting recommends to mention the presence or absence of cribriform architecture in pathology reports [19]. Gleason pattern 5 is composed of tumor cells without glandular differentiation, encompassing single cells, cords and solid fields. The solid field pattern includes large solid areas, small nests, and fields with rosette-like spaces. Additionally, comedonecrosis occurring in cribriform, solid or papillary adenocarcinoma is assigned Gleason pattern 5. At present little is known about the clinical relevance of individual Gleason 5 growth patterns.

Individual growth patterns and cribriform architecture are increasingly considered in risk stratification and clinical decision-making, particularly in men with biopsy Gleason score 3 + 4 = 7 (Grade Group 2) prostate cancer. The presence of cribriform pattern even has prognostic value in Gleason score 8 patients [20–22]. It is yet unclear whether cribriform architecture also has independent predictive value for the highest Gleason scores 9 and 10 (Grade Group 5). The aims of our study were a) to characterize the prognostic value of individual Gleason 5 patterns in a large RP cohort, and b) to assess their impact together with cribriform architecture on clinical outcome in Grade Group 5 prostate cancer patients.

## Methods

### Patient selection

Patients who had undergone RP for prostatic adenocarcinoma from three tertiary medical centers in The Netherlands between 2000 and 2017 were included in this study; 854 patients underwent surgical procedure at Erasmus MC, University Medical Center, Rotterdam; 96 at Leiden University Medical Center (LUMC), Leiden; and 137 at Antoni van Leeuwenhoek Hospital, the Netherlands Cancer Institute (NKI), Amsterdam. Whereas the RPs from Erasmus MC were consecutive specimens, those from LUMC and NKI were selected for presence of Gleason score 4 + 3 to 10 in the original pathology report. We excluded men who had undergone hormonal, radiation, and/or viral therapy (*n* = 23) prior to operation. The specimens had all been fixed in neutral-buffered formalin, sectioned transversely and embedded entirely for diagnostic purposes. All slides were available for pathology review. This study was approved by the institutional Medical Research Ethics Committee (MEC-2018-1614).

### Pathologic evaluation

All RP specimens were reviewed in common sessions by two investigators (EH, GvL), blinded to clinical outcome. For each specimen the following features were recorded: Gleason score and Grade Group according to the 2016 WHO/2014 ISUP guidelines, pT-stage according to the American Joint Committee on Cancer (AJCC) TNM 8th edition, surgical margin status, presence of intraductal carcinoma and Gleason pattern percentages [3, 23].

**Fig. 1:**
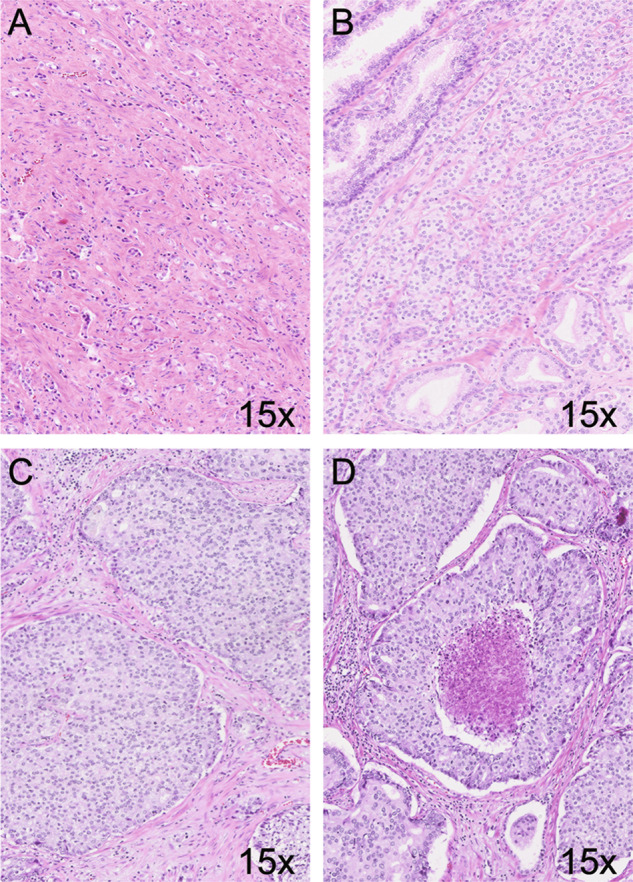
Gleason pattern 5 tumor morphology. **A** Single cells and cords, 15×. **B** Small solid nests, 15×. **C** Medium to large solid fields, 15×. **D** Comedonecrosis, 15×.

The following Gleason 4 growth patterns were recognized: poorly formed, fused, glomeruloid, and cribriform glandular structures [3, 24]. Furthermore, we distinguished small and large cribriform architecture, the latter being defined as having a diameter at least twice the size of adjacent benign glands [25]. The following Gleason 5 growth patterns were identified: single cells, cords, and solid fields (Fig. 1). Single cells and cords were grouped for analysis. Solid fields were arbitrarily categorized as either small solid nests of 10–30 cells, or as medium to large solid fields consisting of more than 30 tumor cells. Rosette-like growth pattern was classified as pattern 4 in case intercellular lumina were recognized and as pattern 5 if they were not present.

Invasive cribriform Gleason pattern 4 and solid pattern 5 either with or without comedonecrosis were morphologically distinguished from intraductal carcinoma based on the following features: invasive cribriform and solid prostate cancer had irregular borders or formed interconnecting fields well exceeding the outline of distended pre-existent glands, or extending into periprostatic adipose tissue, ejaculatory ducts or seminal vesicles. Intraductal carcinoma was continuous with pre-existent glands lined by basal cells or containing corpora amylacea. When invasive cribriform or solid carcinoma and intraductal carcinoma could not be distinguished by morphological criteria alone, additional basal cell immunohistochemistry was performed (22.1% of total cases). If basal cells were completely absent, the lesion was classified as either invasive cribriform Gleason pattern 4 or solid pattern 5 carcinoma. In case sporadic, scattered or continuous basal cells were identified, the lesion was labeled as intraductal carcinoma. In this study, comedonecrosis was considered Gleason pattern 5 when present in invasive cribriform or solid fields, whereas necrosis within intraductal carcinoma did not qualify as Gleason pattern 5. In this study we only considered comedonecrosis in invasive cancer and not in intraductal carcinoma. Intraductal carcinoma and tertiary patterns were not incorporated in the Gleason score [3, 24, 26]. Minor high-grade components occupying <5% of tumor volume were considered as tertiary pattern. The Grade Group concordance rate at revision was 88/135 (65%) for RPs from NKI and 39/94 (41%) for specimens from LUMC.

### Clinical follow-up

Post-operative clinical follow-up consisted of six-monthly and later annual monitoring of serum Prostate-specific Antigen (PSA) levels. Biochemical recurrence was defined as PSA levels ≥0.2 ng/ml measured at two consecutive points in time, at least three months apart with undetectable PSA levels after operation, or as PSA increase of >2.0 ng/ml when serum PSA had not declined to zero after operation. Post-operative lymph node and distant metastases were confirmed by biopsy or multidisciplinary consensus.

### Statistical analysis

Continuous variables were analyzed using the Mann–Whitney *U* test. Pearson’s chi-squared (χ^2^) test was used for the comparison of categorical parameters. Missing PSA values (*n* = 27) were imputed using the median PSA value. Biochemical recurrence-free survival and metastasis-free survival were analyzed using Cox proportional hazards regression and visualized by Kaplan–Meier curves. Statistics were performed using SPSS version 24 (IBM, Chicago, IL, USA). Results were considered significant when the two-sided *P* value was <0.05.

## Results

### Patient characteristics

The entire cohort consisted of 1064 men with a median age of 64.6 years (interquartile range (IQR) 60.2-68.1 years) and median serum PSA level of 8.3 ng/ml (IQR 6.0–13.2 ng/ml). Grade Groups were distributed as follows: 207 (19.4%) Grade Group 1, 472 (44.4%) Grade Group 2, 126 (11.8%) Grade Group 3, 140 (13.2%) Grade Group 4, and 119 (11.2%) Grade Group 5 tumors. Pathologic tumor stage was T2 in 582 (54.7%), T3a in 334 (31.4%) and T3b in 145 (13.6%) patients. Three (0.3%) men had a T4 tumor and were grouped with T3b tumors for further analysis. Positive surgical margins were present in 389 (36.6%) cases. Pelvic lymph node dissection (PLND) was performed in 665 (62.5%) men of whom 63 (9.5%) had metastasis. The median follow-up of the entire cohort (*n* = 1064) was 64 months (IQR 22–105 months). Detailed clinicopathological characteristics are summarized in Table 1.

**Table 1:** Clinicopathological characteristics of the entire cohort (*n* = 1064) stratified for Grade Group (GG).

	*GG1 n* = *207*	*GG2 n* = *472*	*GG3 n* = *126*	*GG4 n* = *140*	*GG5 n* = *119*
Age	62.5 (63.2; 59.8–66.7)	63.8 (64.6; 59.9–68.0)	65.1 (66.2; 60.9–69.9)	64.7 (65.3; 61.4–68.5)	64.7 (64.7; 60.8–70.0)
PSA (ng/ml)	7.0 (6.3; 4.0–9.2)	11.0 (8.3; 6.0–12.9)	16.1 (11.6; 7.2–19.1)	12.9 (10.0; 7.2–16.0)	17.6 (11.3; 7.1–19.0)
pT-stage					
pT2	185 (89%)	268 (57%)	37 (29%)	67 (48%)	25 (21%)
pT3	20 (10%)	169 (36%)	53 (42%)	44 (31%)	48 (40%)
pT3b/T4	2 (1%)	35 (7%)	36 (29%)	29 (21%)	46 (39%)
Positive surgical margin	35 (17%)	156 (33%)	63 (50%)	68 (49%)	67 (56%)
Lymph node status					
Nx	73 (35%)	210 (45%)	39 (31%)	49 (35%)	28 (24%)
N0	134 (65%)	249 (53%)	66 (52%)	79 (56%)	74 (62%)
N1	0	13 (3%)	21 (17%)	12 (9%)	17 (14%)
Cribriform architecture	9 (4%)	252 (53%)	118 (94%)	87 (62%)	102 (86%)
Gleason pattern 5					
Primary				18 (13%)	29 (24%)
Secondary				76 (54%)	90 (76%)
Tertiary	1 (0.5%)	56 (12%)	53 (42%)	16 (11%)	
Single cell/cords	1 (0.5%)	55 (12%)	51 (40%)	99 (71%)	115 (97%)
Small solid nests	0	4 (1%)	2 (2%)	23 (16%)	21 (18%)
Medium to large solid fields	0	2 (0.4%)	1 (1%)	15 (11%)	43 (36%)
Comedonecrosis	0	3 (0.6%)	3 (2%)	9 (6%)	17 (14%)
Biochemical recurrence	16 (8%)	107 (23%)	74 (59%)	68 (49%)	77 (65%)
Distant metastasis	0	18 (4%)	35 (28%)	36 (26%)	47 (39%)
Disease-specific death	0	3 (0.6%)	4 (3%)	12 (9%)	18 (15%)

Values denote either *n* (%) or mean (median; interquartile range).

### Gleason 4 and 5 growth patterns in entire cohort (*n* = 1064)

Cribriform architecture was present in 568 (53.4%) RP specimens. Both intraductal carcinoma and invasive cribriform carcinoma were present in 264 (24.8%) men, while 50 (4.7%) only had intraductal carcinoma and 254 (23.9%) had invasive cribriform but no intraductal carcinoma. Large cribriform carcinoma was observed in 190 (17.9%) men. Any Gleason pattern 5 was recognized in 339 (31.9%) RP specimens, being the primary pattern in 47 (4.4%) tumors, the secondary pattern in 166 (15.6%) and the tertiary pattern in 126 (11.8%) tumors (Table 1). Single cells/cords were identified in 321 (30.2%) RP specimens. Solid fields were present in 90 (8.5%) cases, of which 50 (4.7%) had small solid nests and 61 (5.7%) medium to large solid fields. Comedonecrosis in invasive carcinoma was observed in 32 (3.0%) invasive tumor fields, 17 (1.6%) of which occurred in invasive solid fields and 15 (1.4%) in invasive cribriform structures. The presence of comedonecrosis was the single reason for Gleason pattern 5 assignment in 5 (0.5%) tumors.

Among 126 tumors with tertiary Gleason pattern 5, single cells/cords were observed in 113 (89.7%) cases and represented the only Gleason 5 pattern in 107/126 (84.9%) men. Single cells/cords coexisted with other Gleason 5 growth patterns in tumors with primary or secondary Gleason pattern 5 in 124/213 cases (58.2%; *P* < 0.001).

Of 339 tumors with Gleason pattern 5, cribriform architecture was present in 245 (72.3%) and absent in 94 (27.7%) cases. No difference was found in presence of single cells/cords between cribriform-negative (91/94, 96.8%) and cribriform-positive (226/245, 92.2%; *P* = 0.13) cases. The presence of small solid nests also did not differ between cribriform-negative (17/94, 18.1%) and cribriform-positive (33/245, 13.5%; *P* = 0.28) cases. In contrast, all 61 men with medium to large solid fields and 32 cases with comedonecrosis also had concomitant cribriform architecture.

### Growth patterns in Grade Group 5 prostate cancer (*n* = 119)

Individual growth patterns were specifically analyzed in 119 men with Grade Group 5 disease, which had Gleason pattern 5 as primary or secondary pattern by definition. This group encompassed 90 (75.7%) men with Gleason score 4 + 5, 28 (23.5%) with 5 + 4 and 1 (0.8%) with 5 + 5. Cribriform architecture was present in 102 (85.7%) and large cribriform growth in 52 (43.7%) tumors. Single cells/cords were present in 115 (96.6%), small solid nests in 21 (17.6%), medium to large solid fields in 43 (36.1%) and comedonecrosis in 17 (14.2%) cases. Comedonecrosis occurred within medium to large solid fields in 12 (10.1%) cases and in invasive cribriform carcinoma in 5 (4.5%) tumors. Out of 102 Grade Group 5 patients with cribriform architecture, 43 (42.2%) had concomitant medium to large solid fields and 59 (57.8%) did not. All tumors with medium to large solid fields and comedonecrosis were accompanied by cribriform architecture. Clinicopathological parameters of Grade Group 5 patients are shown in Table 2.

**Table 2:** Characteristics of Grade Group 5 patients (*n* = 119) stratified for presence of cribriform and solid growth patterns.

	Cribriform negative Solid negative *n* = 17	*P* value^a^	Cribriform positive Solid negative *n* = 59	*P* value^b^	Cribriform positive Solid positive *n* = 43
PSA (ng/ml)	10.1 (8.2; 6.3–13.2)	0.19	18.4 (9.8; 6.3–22.0)	0.31	19.7 (12.0; 7.8–20.0)
pT-stage					
pT2	9 (53%)	0.001	7 (12%)	0.32	9 (21%)
pT3a	5 (29%)		24 (41%)		19 (44%)
pT3b/T4	3 (18%)		28 (47%)		15 (35%)
Positive surgical margin	8 (47%)	0.19	38 (64%)	0.12	21 (49%)
Lymph node status					
Nx	5 (29%)	0.15	13 (22%)	0.82	10 (23%)
N0	12 (71%)		35 (59%)		27 (63%)
N1	0		11 (19%)		6 (14%)
Biochemical recurrence^c^	2 (12%)	<0.001	43 (73%)	0.98	32 (74%)
Distant metastasis^c^	0	0.005	22 (37%)	0.20	25 (58%)
Disease-specific death^c^	0	0.15	8 (14%)	0.40	10 (23%)

^a^Cribriform negative/Solid negative *versus* Cribriform positive/Solid negative.

^b^Cribriform positive/Solid negative *versu*s Cribriform positive/Solid positive.

^c^*P* values refer to log-rank analysis.

**Fig. 2:**
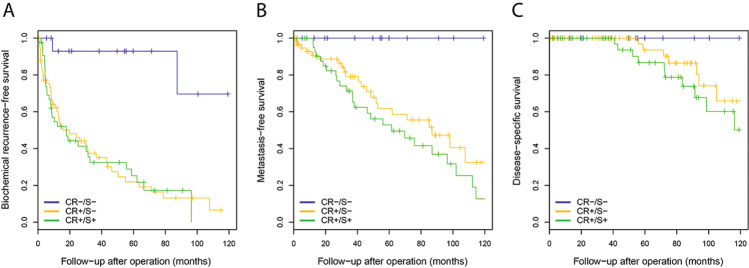
Clinical outcome of Grade Group 5 prostate cancer patients without cribriform and solid pattern (CR−/S−; blue line, *n* = 17), with cribriform but no solid pattern (CR+/S−; orange line, *n* = 59), and with both cribriform and solid pattern (CR+/S+; green line, *n* = 43). Grade Group 5 patients without cribriform and solid pattern (CR−/S−) had significantly better biochemical recurrence-free survival (**A**; log-rank *P* < 0.001) and, metastasis-free survival (**B**, log-rank *P* = 0.003), while disease-specific survival did not reach significance (**C**; log-rank *P* = 0.18).

Grade Group 5 patients with cribriform architecture had significantly more frequent non-organ confined disease (pT3/4 86/102, 84.3%) than men without cribriform pattern (8/17, 47.1%; *P* = 0.002). While the first group also had higher PSA levels (18.8 ng/ml *versus* 10.1 ng/ml, *P* = 0.12) and more PLND metastasis (17/78; 21.8% *versus* 0/12; 0%, *P* = 0.07) than the latter, this did not reach conventional levels of significance putatively due to a limited number of cases. Biochemical recurrence-free survival was significantly shorter for men with cribriform architecture (log-rank *P* < 0.001; Fig. 2). None of the Grade Group 5 patients without cribriform architecture developed post-operative metastasis or died from disease, while 47 (47/102, 46.1%; log-rank *P* = 0.002) and 18 (18/102, 17.6%; log-rank *P* = 0.10) men with cribriform architecture did, respectively.

Among Grade Group 5 patients with cribriform architecture, those with medium to large solid fields had comparable PSA levels (19.7 *versus* 18.4 ng/ml; *P* = 0.31), non-organ confined disease (pT3/4 34/43, 79.1% *versus* 52/59, 88.1%; *P* = 0.32), positive surgical margins (21/43, 48.8% *versus* 38/59, 64.4%; *P* = 0.12) and PLND metastasis (6/33, 18.2% *versus* 11/46, 23.9%; *P* = 0.54) to men without medium to large solid fields. Likewise, post-operative biochemical recurrence-free (log-rank *P* = 0.98), metastasis-free (log-rank *P* = 0.20) and disease specific-free (log-rank *P* = 0.40) survival did not differ between cribriform Grade Group 5 men with and without medium to large solid fields (Fig. 2).

### Biochemical recurrence- and metastasis-free survival

Biochemical recurrence occurred in 342 (32.1%) men after a median of 17 months. Biochemical recurrence rates were 16/207 (7.7%) for Grade Group 1, 107/472 (22.7%) for Grade Group 2, 74/126 (58.7%) for Grade Group 3, 68/140 (48.6%) for Grade Group 4 and 77/119 (64.7%) for Grade Group 5. In multivariable analysis, the presence of cribriform architecture (Hazard Ratio (HR) 2.1; 95% Confidence Interval (CI) 1.5–2.9; *P* < 0.001) and comedonecrosis (HR 2.1; 95% CI 1.3–3.2; *P* = 0.001) were independently associated with biochemical recurrence, as were PSA, Grade Group, pT-stage, positive surgical margins and lymph node status, while single cell/cords, small solid nests and medium to large solid fields were not (Table 3).

**Table 3:** Multivariable Cox regression analysis for biochemical recurrence-free survival and metastasis-free survival after radical prostatectomy in the entire cohort (*n* = 1064).

	Biochemical recurrence			Metastasis		
	HR	95% CI	*P* value	HR	95% CI	*P* value
Log(2) PSA (ng/ml)	1.3	1.1–1.4	<0.001	0.9	0.8–1.1	0.17
pT-stage						
T2	*ref*			*ref*		
T3a	1.7	1.3–2.3	<0.001	1.5	0.9–2.5	0.08
T3b/T4	1.9	1.4–2.7	<0.001	1.4	0.8–2.5	0.20
Grade Groups						
1	*ref*			*ref*^a^		
2	1.8	1.0–3.3	0.04			
3	3.3	1.7–6.2	<0.001	3.9	2.1–7.4	<0.001
4	3.8	2.0–7.2	<0.001	6.0	3.1–11.6	<0.001
5	4.1	2.0–8.2	<0.001	5.2	2.4–11.1	<0.001
Positive surgical margin	1.6	1.3–2.0	<0.001	1.3	0.9–1.8	0.24
Pelvic lymph node status						
Nx	*ref*			*ref*		
N0	1.4	1.1–1.8	0.01	1.6	1.0–2.6	0.04
N1	3.8	2.6–5.7	<0.001	4.4	2.5–7.6	<0.001
Cribriform architecture	2.1	1.5–2.9	<0.001	9.9	3.9–25.5	<0.001
Single cell/cords	0.9	0.6–1.2	0.39	1.2	0.7–1.8	0.55
Small solid nests	0.9	0.6–1.4	0.64	0.6	0.3–1.2	0.13
Medium to large solid fields	0.9	0.6–1.4	0.63	1.6	0.9–2.7	0.09
Comedonecrosis	2.1	1.3–3.2	0.001	2.1	1.2–3.7	0.01

*HR* hazard ratio, *CI* confidence interval.

^a^Grade Group 1 (no events) and 2 are grouped for metastasis.

Post-operative lymph node and distant metastases were observed in 136 (12.7%) men after a median of 47 months: 0/207 (0%) in Grade Group 1, 18/472 (3.8%) in Grade Group 2, 35/126 (27.8%) in Grade Group 3, 36/140 (25.7%) in Grade Group 4 and 47/119 (39.5%) in Grade Group 5. Since no metastasis occurred in Grade Group 1 patients, we grouped those patients with Grade Group 2 in further analysis. Multivariable analysis demonstrated that cribriform architecture (HR 9.9; 95% 3.9–25.5; *P* < 0.001) and comedonecrosis (HR 2.1; 95% 1.2–3.7; *P* = 0.01) had independent predictive value for metastasis-free survival together with Grade Groups and lymph node status; in this model PSA, pT-stage, positive surgical margin status and single cell/cords did not have independent predictive value (Table 3). Medium to large solid fields showed a trend towards unfavorable outcome, however this did not reach statistical significance (*P* = 0.09). Disease-specific death occurred in 37 (3.5%) men after a median of 72 months: 0/207 (0%) in Grade Group 1, 3/472 (0.6%) in Grade Group 2, 4/126 (3.2%) in Grade Group 3, 12/140 (8.6%) in Grade Group 4 and 18/119 (15.1%) in Grade Group 5 tumors. The number of events was too small for multivariable statistical analysis.

## Discussion

In this study, we analyzed the histomorphology of Gleason grade 5 growth patterns and their impact on clinicopathological outcome. Gleason pattern 5 was present in 32% of prostate cancer specimens. Single cells/cords were the most common pattern, being present in 95% of Gleason pattern 5 positive tumors. Small solid nests, medium to large solid fields and comedonecrosis in invasive carcinoma were seen in 15%, 18% and 9% of tumors with Gleason pattern 5, respectively. All men with medium to large solid fields or comedonecrosis had concomitant cribriform architecture, an association not found for single cells/cords or small solid nests. Cribriform architecture and presence of comedonecrosis were independent predictive parameters for biochemical recurrence- and metastasis-free survival, while single cells/cords, small solid nests and medium to large solid fields growth patterns were not. Our results demonstrate that comedonecrosis in invasive prostate cancer is an independent parameter for disease outcome and that cribriform architecture has independent predictive value even in Grade Group 5 patients.

Our findings on the occurrence and prognostic impact of Gleason 5 growth patterns are in line with previous studies. Single cells/cords are the most common Gleason 5 pattern [27, 28]. In a study of 49 Grade Group 5 RPs, comedonecrosis, sheets and solid Gleason pattern 5 were associated with biochemical recurrence in univariate analysis, while single cells/cords were not [27]. In a RP cohort, Acosta et al. found that comedonecrosis was often concurrently present in intraductal and invasive carcinoma, and that within invasive carcinoma in high-grade prostate cancer it was associated with adverse pathologic features and biochemical recurrence [29, 30]. In accordance with these studies, our findings indicate that comedonecrosis in invasive Gleason pattern 5 is associated with more aggressive features than single cells/cords and solid fields, even when cribriform architecture is taken into account. Comedonecrosis occurs more frequently in intraductal than invasive carcinoma [31]. In our study we only monitored comedonecrosis in invasive carcinoma as its presence in intraductal carcinoma is not graded according to the 2014 ISUP recommendations [3]. For future studies it is of interest to determine whether men with comedonecrosis in intraductal carcinoma also have worse outcome compared to those without comedonecrosis in intraductal carcinoma.

In general presence of invasive cribriform and/or intraductal carcinoma has independently been associated with adverse pathologic features and shorter biochemical recurrence-free, metastasis-free and disease specific-free survival [17, 32]. While most of these studies were performed in men with Gleason score 7 prostate cancer, cribriform architecture also is an independent parameter for biochemical recurrence and distant metastasis in Gleason score 8 patients [20–22]. In a biopsy screening cohort Kweldam et al. found that Grade Group 5 patients with cribriform architecture had significantly shorter disease-specific survival than men without this pattern [21]. In the present study, we confirmed this for RP specimens. None of the 17 Grade Group 5 men without cribriform architecture developed post-operative metastasis, while 46% of men with cribriform architecture did. We demonstrate that even in the most aggressive Grade Group absence of cribriform architecture is associated with better outcome.

Besides clinical relevance, our findings provide comprehensive insight in the biological relation of prostate cancer growth patterns. Previously, Verhoef et al. made detailed three-dimensional reconstructions of prostate cancer architectural structures and defined two major groups of growth patterns [33]. The first group encompassed Gleason pattern 3, poorly formed and fused pattern 4, and single cells/cords pattern 5. These patterns form a continuum of interconnecting tubules with increased branching and decreasing luminal diameter in higher grades, which have in common that the vast majority of tumor cells directly contact adjacent stroma. The second group includes cribriform Gleason pattern 4 and solid pattern 5, both either with or without comedonecrosis. These patterns also form a continuum of contiguous tumor cells in which the vast majority of cells does not have direct contact with the surrounding stroma. The findings of the current study are completely in line with this proposed growth pattern model. Medium to large solid field pattern 5 was always observed with cribriform architecture, indicating their strong morphological relationship. On the other hand, single cells/cords pattern 5 also occurred without cribriform or solid fields, and was never found to be spatially continuous with these patterns (*data not shown*), indicating their different morphogenesis. Furthermore, the distinction between these growth patterns is also reflected by the worse clinical outcome of patients with cribriform architecture. The coexistence and proposed morphological continuum of medium to large solid fields and cribriform architecture could explain the association between solid fields and unfavorable clinical outcome. The occurrence of comedonecrosis might point towards accelerated growth or metabolic aberrations being associated with more aggressive biological behavior of cribriform and solid carcinoma. Chua et al. demonstrated that cribriform architecture was associated with genomic instability and hypoxia [34]. Comedonecrosis could be regarded as a morphological end-stage of hypoxia, comprehensively linking three-dimensional morphogenesis, clinical outcome, and hypoxia. Invasive cribriform and solid carcinoma are associated with *c-MYC* amplification, loss of *PTEN* and *SPOP* point mutations [34–38]. These molecular aberrations have been linked to aggressive clinical behavior in prostate cancer and provide a rationale for the dismal outcome of patients with these growth patterns [39–45].

In the current study, we distinguished small solid nests from medium to large solid fields. While the latter were clearly associated with cribriform pattern, the former pattern was not. Thirty-four percent of patients with small solid nests did not have cribriform architecture; small solid nests were not independently associated with worse outcome and not spatially continuous with cribriform architecture. At present, it is not clear in what morphogenetic continuum small solid nests belong. Based on the above-mentioned mutual relation of growth patterns, we hypothesize that small solid nests might be precursor lesions of fused glands prior to lumen-formation. Interestingly, Shah et al. found overall consensus among 16 urologic pathologists for calling large (>20 cells) solid fields Gleason pattern 5, but not for medium (10–20 cells) or small (<10 cells) solid nests, further questioning the precise clinicopathologic relevance of smaller sized solid fields [46].

Strong points of this study are the detailed histological review of all RP specimens and the classification of individual Gleason growth pattern using strict histomorphological criteria and additional immunohistochemistry. However, the study is limited by the retrospective study design, relatively low number of patients with high-grade disease and heterogeneity of the study population including selected patients from two centers and not accounting for adjuvant therapy.

In conclusion, we demonstrate that comedonecrosis in invasive cancer is an independent predictive Gleason 5 pattern for biochemical recurrence- and metastasis-free survival after RP, while single cells/cords, small solid nest and medium to large solid fields are not. Furthermore, cribriform architecture has additional value for clinical outcome among Grade Group 5 prostate cancer patients. On the other hand, Grade Group 5 men without cribriform architecture have relatively good outcome. These data support the current recommendations to routinely include cribriform pattern in pathology reports and indicate that presence of comedonecrosis in invasive cancer should also be commented on.

## Data Availability

The datasets generated and/or analyzed during the current study are not publicly available due to patient confidentiality but are available (anonymized) from the corresponding author on reasonable request.
